# Supplementary material for: HIVprotI: an integrated web based platform for prediction and design of HIV proteins inhibitors
Source: J Cheminform. 2018 Mar 9;10:12. doi: 10.1186/s13321-018-0266-y (PMC5845081; doi:10.1186/s13321-018-0266-y)
Supplement: Supplementary file 2 — Additional file 2. Source code of HIVProtI web server. [file 13321_2018_266_MOESM2_ESM.zip › HIVprotI_Source-code/hivprotI/weka-3-6-6/doc/weka/classifiers/bayes/net/estimate/DiscreteEstimatorFullBayes.html]

DiscreteEstimatorFullBayes


---


|  |  |  |
| --- | --- | --- |
| **Overview** \| **Package** \| **Class** \| **Tree** \| **Deprecated** \| **Index** \| **Help** \| **Weka's home** | |  |
| **PREV CLASS**   **NEXT CLASS** | **FRAMES**    **NO FRAMES**     **All Classes** |  |
| SUMMARY: NESTED \| FIELD \| CONSTR \| METHOD | DETAIL: FIELD \| CONSTR \| METHOD |  |


---


## weka.classifiers.bayes.net.estimate Class DiscreteEstimatorFullBayes

```
java.lang.Object
  weka.estimators.Estimator
      weka.classifiers.bayes.net.estimate.DiscreteEstimatorBayes
          weka.classifiers.bayes.net.estimate.DiscreteEstimatorFullBayes
```

**All Implemented Interfaces:**: java.io.Serializable, java.lang.Cloneable, Scoreable, CapabilitiesHandler, OptionHandler, RevisionHandler

---

``` public class DiscreteEstimatorFullBayes extends DiscreteEstimatorBayes ```

Symbolic probability estimator based on symbol counts and a prior.

**Version:**
:   $Revision: 1.3 $

**Author:**
:   Remco Bouckaert (rrb@xm.co.nz)

**See Also:**: Serialized Form

---

**Field Summary**

**Fields inherited from interface weka.classifiers.bayes.net.search.local.Scoreable**

`AIC, BAYES, BDeu, ENTROPY, MDL`


**Constructor Summary**

`DiscreteEstimatorFullBayes(int nSymbols,
double w1,
double w2,
DiscreteEstimatorBayes EmptyDist,
DiscreteEstimatorBayes ClassDist,
double fPrior)`
  
          Constructor


| **Method Summary** | |
| --- | --- |
| `java.lang.String` | `getRevision()`             Returns the revision string. |
| `static void` | `main(java.lang.String[] argv)`             Main method for testing this class. |

**Methods inherited from class weka.classifiers.bayes.net.estimate.DiscreteEstimatorBayes**

`addValue, getCount, getNumSymbols, getProbability, logScore, toString`

**Methods inherited from class weka.estimators.Estimator**

`addValues, addValues, addValues, addValues, buildEstimator, buildEstimator, clone, debugTipText, equals, forName, getCapabilities, getDebug, getOptions, listOptions, makeCopies, makeCopy, setDebug, setOptions, testCapabilities`

**Methods inherited from class java.lang.Object**

`getClass, hashCode, notify, notifyAll, wait, wait, wait`

**Constructor Detail**

### DiscreteEstimatorFullBayes

```
public DiscreteEstimatorFullBayes(int nSymbols,
                                  double w1,
                                  double w2,
                                  DiscreteEstimatorBayes EmptyDist,
                                  DiscreteEstimatorBayes ClassDist,
                                  double fPrior)
```

:   Constructor

    **Parameters:**: `nSymbols` - the number of possible symbols (remember to include 0): `w1` -: `w2` -: `EmptyDist` -: `ClassDist` -: `fPrior` -


**Method Detail**

### getRevision

```
public java.lang.String getRevision()
```

:   Returns the revision string.

    :   **Specified by:**: `getRevision` in interface `RevisionHandler` **Overrides:**: `getRevision` in class `DiscreteEstimatorBayes`
    :   **Returns:**: the revision

---


### main

```
public static void main(java.lang.String[] argv)
```

:   Main method for testing this class.

    :   **Parameters:**: `argv` - should contain a sequence of integers which will be treated as symbolic.


---


|  |  |  |
| --- | --- | --- |
| **Overview** \| **Package** \| **Class** \| **Tree** \| **Deprecated** \| **Index** \| **Help** \| **Weka's home** | |  |
| **PREV CLASS**   **NEXT CLASS** | **FRAMES**    **NO FRAMES**     **All Classes** |  |
| SUMMARY: NESTED \| FIELD \| CONSTR \| METHOD | DETAIL: FIELD \| CONSTR \| METHOD |  |


---
